# Workshop on Blood Loss Quantification in Obstetrics: Improving Medical Student Learning through Clinical Simulation

**DOI:** 10.3390/healthcare10020399

**Published:** 2022-02-21

**Authors:** Javier Ruiz-Labarta, Ana Martínez Martín, Pilar Pintado Recarte, Blanca González Garzón, Juan Manuel Pina Moreno, Mercedes Sánchez Rodríguez, África Vallejo Gea, Luis Sordo, Melchor Álvarez-Mon, Miguel A. Ortega, Coral Bravo Arribas, Juan A. De León-Luis

**Affiliations:** 1 Department of Public and Maternal and Child Health, School of Medicine, Complutense University of Madrid, 28040 Madrid, Spain; franciscojavier.ruiz@salud.madrid.org (J.R.-L.); anmart22@ucm.es (A.M.M.); ppintado@salud.madrid.org (P.P.R.); bgonzalezg@salud.madrid.org (B.G.G.); juanmanuel.pina@salud.madrid.org (J.M.P.M.); mariamercedes.sanchez.rodriguez@salud.madrid.org (M.S.R.); africava@ucm.es (Á.V.G.); lsordo@ucm.es (L.S.); jaleon@ucm.es (J.A.D.L.-L.); 2 Department of Obstetrics and Gynecology, University Hospital Gregorio Marañón, 28009 Madrid, Spain; 3 Health Research Institute Gregorio Marañón, Consortium for Biomedical Research in Epidemiology and Public Health (CIBERESP), 28009 Madrid, Spain; 4 Maternal and Infant Research Investigation Unit, Alonso Family Foundation (UDIMIFFA), 28009 Madrid, Spain; 5 Department of Medicine and Medical Specialties, Faculty of Medicine and Health Sciences, University of Alcalá, 28801 Madrid, Spain; mademons@gmail.com; 6 Ramón y Cajal Institute of Healthcare Research (IRYCIS), 28034 Madrid, Spain; 7 Immune System Diseases-Rheumatology and Oncology Service, University Hospital Príncipe de Asturias, CIBEREHD, 28805 Alcalá de Henares, Spain

**Keywords:** clinical simulation, blood loss quantification, obstetric haemorrhage, knowledge, self-assurance, self-confidence, usefulness, feedback

## Abstract

Purpose: To assess whether a clinical simulation-based obstetric blood loss quantification workshop for medical undergraduate trainees improves theoretical–practical knowledge, along with self-assurance and self-confidence. Methods: This was a quasi-experimental pre-post learning study conducted at the Gynaecology and Obstetrics Unit of the Hospital Gregorio Marañón, Madrid, Spain. Participants were volunteer students in their fourth year of a 6-year degree course in Medicine. The study period was divided into the stages: pre-workshop, intra-workshop, 2 weeks post-workshop and 6 months post-workshop. In the pre-workshop stage, students completed a brief online course in preparation for the workshop. The effectiveness of the workshop was assessed through multiple choice tests and self-administered questionnaires. Data were compared between time-points using statistical tests for paired samples. Results: Of the 142 students invited (age 21.94 ± 3.12 years), 138 accepted the offer of the workshop (97.2%), and 85.4% had no experience in managing blood loss. Between the stages pre- and 2 weeks post-workshop, significant improvements were observed in theoretical–practical knowledge (μ = 1.109), self-assurance and self-confidence. At the 6 months post-workshop stage, theoretical–practical knowledge diminished compared with 2 weeks post-workshop, returning to pre-workshop levels, while self-assurance and confidence failed to vary significantly in the longer term. Conclusions: The obstetric workshop improved theoretical–practical knowledge and the self-assurance and confidence of the medical students. Results 2 weeks post-workshop were maintained up until 6 months after the training intervention. The clinical simulation-based workshop was perceived by the students as useful and necessary.

## 1. Introduction

In the past decade, clinical simulation as a learning method for medical undergraduates has advanced tremendously at universities and teaching hospitals across the world. Clinical simulation is the fictitious performance of a complex clinical procedure with sufficient realism to facilitate the acquisition of theoretical–practical skills, including communication and coordination with medical staff, through immersion, practice and feedback, while avoiding risks inherent to real healthcare situations. Among others, its benefits are learning curve shortening, improved patient confidence and competitive results [1].

Since 2017, the Gynaecology and Obstetrics Unit of the Hospital Universitario Gregorio Marañón (HUGM), Madrid, Spain, has been holding a series of clinical simulation workshops for undergraduates of medicine from the Universidad Complutense de Madrid (UCM), aimed at improving their understanding of maternal–neonatal health. These workshops are popular and seem subjectively to improve the theoretical–practical skills and self-assurance and confidence of participants. However, to date, no effort has been made to assess their effectiveness in an objective manner.

This study sought to assess the results of the workshop on “Quantification of blood loss in Obstetrics” conducted in 2020–2021 with the participation of students in their fourth year of a 6-year degree course in Medicine. The hospital teaching staff selected this workshop on the grounds of the importance of adequately managing obstetric haemorrhage [2,3]. There is no consensus in the literature when it comes to defining postpartum haemorrhage; however, one of the most accepted definitions is the one that defines it as a blood loss that exceeds 500 mL after a vaginal delivery, or 1000 mL if it is a caesarean section9. Postpartum haemorrhage appears in 1–5% of deliveries in our setting.

This complication of childbirth is the leading cause of maternal mortality, both in emergent and industrialized countries [4,5]. The workshop strives to teach students the necessary tools to learn how to diagnose an obstetric haemorrhage through the gravimetric quantification of blood loss in different settings (postpartum or during a caesarean birth).

The HUGM is a tertiary hospital that serves patients with special risk of the appearance of obstetric haemorrhage (induced labour, caesarean birth, twin birth or older maternal age). Our department is currently working towards instructing all healthcare workers on how to quantify blood loss by weighing fluids and sterile gauze during every delivery, in an attempt to reduce maternal morbidity and mortality. We also have an established multidisciplinary protocol for severe obstetric haemorrhage to help with the prevention, diagnosis and treatment of patients with this complication of childbirth [6,7].

Visual estimation of obstetric-related blood loss alone is poorly sensitive and specific, as it tends to underestimate real blood loss [3]. Thus, a more objective and accurate method of estimating excessive bleeding is needed [8]. Gravimetric quantification consists of weighing fluid losses collected in a calibrated under-buttocks drape or suction canister and adding this volume to that measured by weighing blood-soaked items or gauzes. This is the preferred method, recommended by guidelines issued by many national and international institutions, such as the American College of Obstetricians and Gynaecologists [9,10]. In effect, many studies have shown that this tool improves the skills and confidence of healthcare professionals [11,12].

The present study was designed to assess if the “Quantification of blood loss in Obstetrics workshop” (hereafter “the workshop”) offered to medical students is useful in terms of improving theoretical–practical knowledge, along with the self-assurance and self-confidence of students when managing blood loss postpartum or during a caesarean delivery.

## 2. Materials and Methods

The study design was quasi-experimental pre–post, with longitudinal follow up from just before to several months after participating in the workshop implemented at the Gynaecology and Obstetrics Dept. of the HUGM, during the academic year 2020–2021. The recruited participants were 4th-year medical students enrolled in the subject of Obstetrics and Gynaecology, who were offered the workshop as part of their practical training in the subject, and who were able to attend voluntarily. The author MM. was in charge of controlling the lists of participants and verifying the correct completion of the tests and questionnaires that they had to fill out through a virtual platform. Later, they were downloaded in order to analyse the answers.

**Figure 1:**
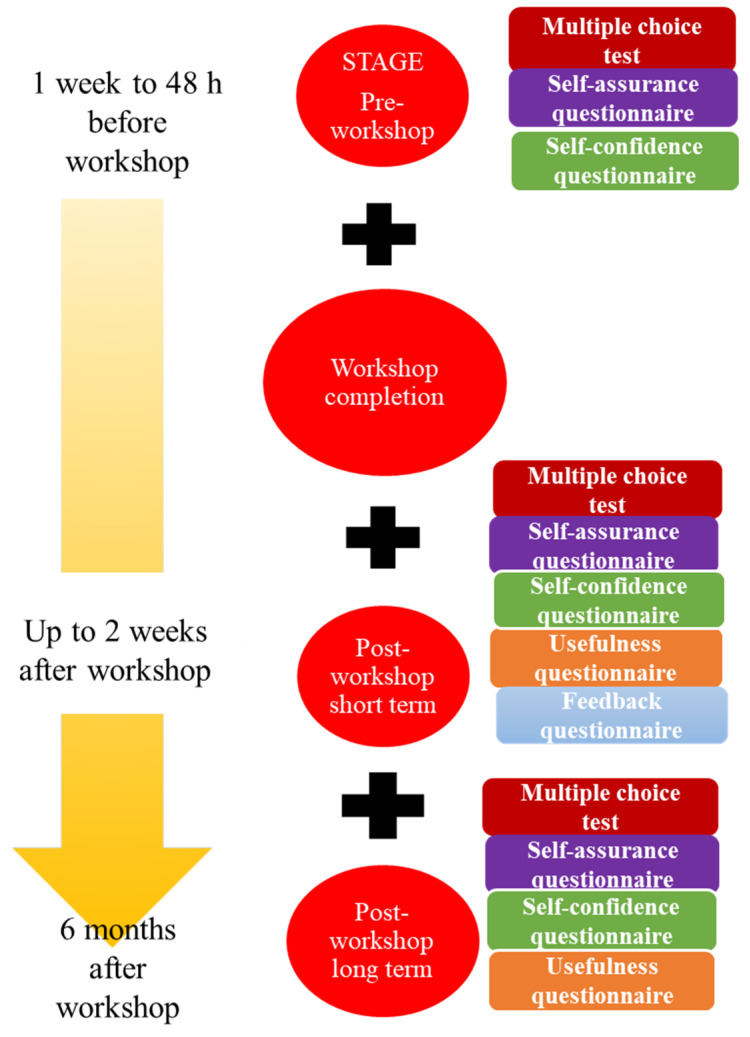
Stages and content of the blood loss quantification workshop.

The workshop was divided into four consecutive stages which were described previously to the students (Figure 1). The stages were: (1) pre-workshop, (2) workshop, (3) up to 2 weeks after the workshop (short term), and (4) 6 months after the workshop (long term):

1. Stage 1: This was executed “*on-line*” via a virtual platform (www.aleesca.es/moodle, accessed on 18 August 2021). Here, the students had access to descriptions of the workshop along with the theory (presentations and videos) related to blood loss quantification in obstetrics. The tasks to be completed were:
  A. Multiple choice test (MCT), in which 20 questions should be answered in 30 min to assess theoretical–practical knowledge pertaining to the subject. For each correct answer, 0.5 points were added (no points were subtracted for incorrect answers).
  B. Two self-administered questionnaires to assess the self-assurance and self-confidence of the students when facing a similar clinical situation. Replies were scored according to a semiquantitative Likert scale [13] (Appendix A).
2. Stage 2: This was the actual clinical simulation workshop completed. Over a period of 1.5 hours, the students, in groups of 8–10, were given a brief lecture on how to quantify blood loss postpartum or during a caesarean section. The students then put their understanding of the topic into practice in different clinical scenarios with the help of a mannequin and artificial blood. Students were encouraged to ask questions during the task. A clinical scenario of a patient who had experienced postpartum haemorrhage after a normal vaginal delivery was depicted. To do this, a mannequin in the shape of a female pelvis was used, with a plastic blood collection bag located under the pelvis and textile material (compresses, gauze pads and underpads) soaked in blood. The student had to perform a gravimetric quantification of the blood lost by the patient during the immediate postpartum period. Subsequently, the students were able to design other simulated clinical scenarios (caesarean section, instrumental delivery) with the same material, to continue practicing gravimetric quantification in other situations.
3. Stages 3 and 4: These stages were completed on-line and included tasks such as:
  C. A similar MCT to that of stage 1, but with questions designed to compare the student’s understanding of the topic and practical skills before and at two time points after the workshop.
  D. Three self-administered questionnaires designed to assess their self-assurance, self-confidence and perception of usefulness of the workshop, and to gain feedback (Appendix A).

Assistance and completion of each workshop stage were noted, so that students missing some of the tasks could be withdrawn from the study. Other exclusion criteria were: students completing the MCT in under 3 min or over 30 min, and those needing more than one attempt at the test.

The variables analysed were (Table 1): sex, age, prior experience, theoretical–practical knowledge, self-assurance, self-confidence, perception of workshop usefulness and feedback.

Replies to the MCT and questionnaires were collected online and transferred to an Excel sheet for their analysis. Statistical tests were performed using the software package SPSS Version 21.0 (IBM Corp., Armonk, NY, USA). Quantitative variables were expressed as the mean ± standard deviation, and categorical variables as their number and percentage. To assess the changes produced in theoretical–practical knowledge (assessed through MCT) between the different stages, we used the Student *t*-test for paired samples with significance set at *p* < 0.05. These statistical tests were also used to assess changes in self-assurance and self-confidence between the different study stages (assessed through self-administered questionnaires). The Kolmogorov–Smirnov test was used to check the normality of the data.

## 3. Results

**Figure 2:**
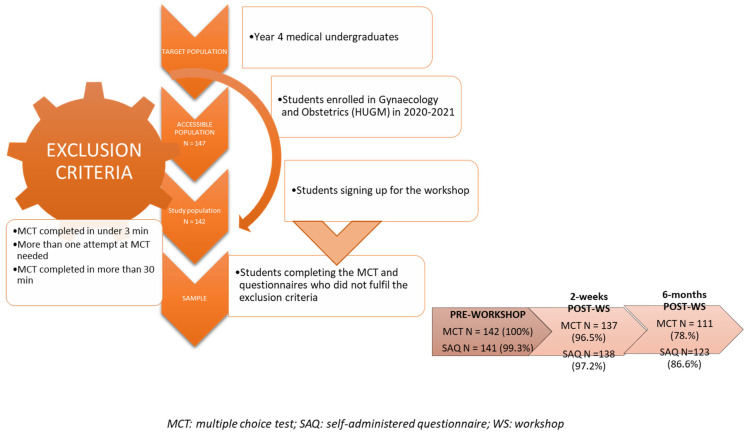
Participant recruitment.

**Figure 3:**
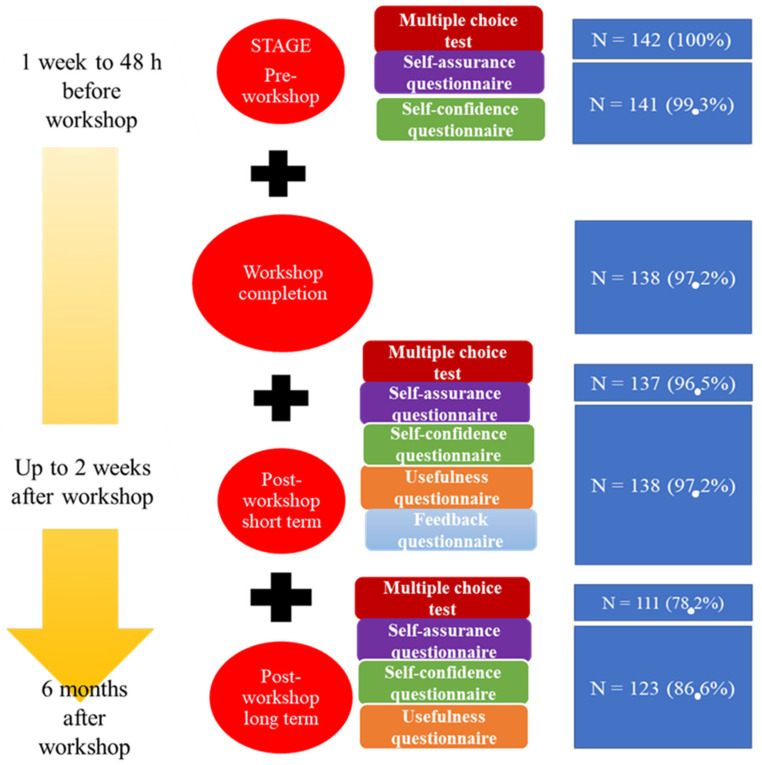
Workshop stages, tasks and participation.

Of the 147 students enrolled in the subject, 142 (97.2%) took part in the workshop. After applying the exclusion criteria, the rate of participation was high (>95%), both at the pre-workshop and workshop stages, and thereafter dropped slightly in the long-term post-workshop stage (78.2%) (Figure 2 and Figure 3).

The mean age of the students was 21.94 ± 3.12 years. Scores obtained in the MCT were high, both in the pre- (mean = 7.47 out of 10) and post-workshop stages (8.52 and 7.47 at 2 weeks and 6 months, respectively). Between the stages pre-workshop and 2 weeks post-workshop, a significant improvement was observed in theoretical–practical knowledge (*p* < 0.05). At 6 months post-workshop, pre-workshop scores in response to the on-line course were maintained (Table 2 and Table 3).

Results for self-reported confidence were similar. A significant improvement in scores was observed between pre-workshop and 2 weeks post-workshop (*p* < 0.05). Between the short- and long-term post-workshop stages, results remained practically stable, with a slight non-significant decrease observed at 6 months (Table 4).

**Figure 4:**
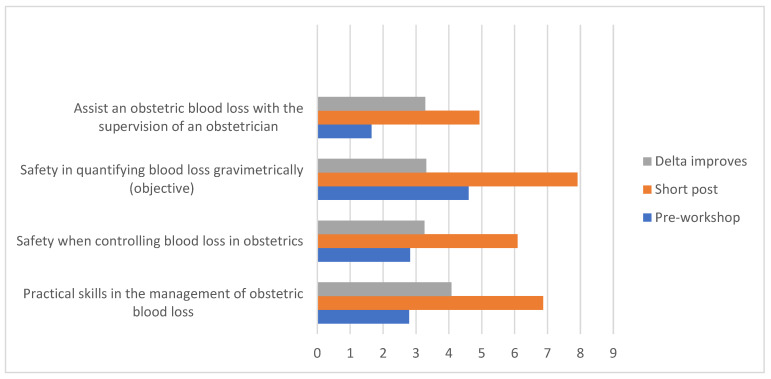
Aptitudes returning the greater improvement delta for pre-workshop vs. 2 weeks post-workshop.

When asked about the workshop’s utility, in both the short and long term after the workshop, close to 90% of the participants considered it useful, this type of intervention being perceived as an essential part of medical training (Table 5, Figure 4).

## 4. Discussion

Initially, 142 medical students signed up for the simulation-based workshop on blood loss quantification in Obstetrics. Participation was high in most study stages (>95%), although this proportion decreased to 78.2% in the long-term stage after the workshop. Theoretical and practical knowledge assessed in the MCT improved significantly from the pre- to short-term post-workshop stages, although this improvement did not persist over time. The self-assurance and confidence of the students also showed significant improvement from before to 2 weeks after the workshop, and these two improvements continued in the long term. The questionnaire designed to assess how useful the workshop was perceived by the students was clear in indicating that they found it useful and necessary for their training.

Despite the high workload involving three MCTs and nine questionnaires to be completed over 6 months, the participation rate in all consecutive stages of this study was high. This high participation highlights how receptive our medical students were to simulation-based training extending for a period of 6 months. The teaching unit of the HUGM hosts a large number of students every year. Furthermore, the division of the workshop into several stages offers continuity in the follow up of students, and the “*on-line*” platform (www.aleesca.es/moodle, accessed on 18 August 2021) offers rapid easy access to the teaching material. Other studies that have assessed a simulation-based approach to blood loss quantification in Obstetrics include one conducted in 44 medicine undergraduates [14], and another one in a setting of midwifery with 65 participants [15]. These studies, nevertheless, had different objectives and procedures than our investigation.

According to the MCT results regarding the acquisition of theoretical–practical knowledge, we should mention that starting from a high mean pre-workshop score of 7.47, this score went up significantly in the short term by one point, and thereafter returned to baseline values at 6 months post-workshop. While this increase may seem unsurprising, we should not forget that the students maintained a high grade (7–9 out of 10) throughout the process, even in the long term, when they had not recently revised the theory of blood loss quantification. Certainly, the study proves the necessity to organize similar repeated training, since the results obtained before the workshop and after 6 months are almost identical (7.47 ± 1.66 vs. 7.47 ± 1.24). This highlights the importance of periodic training to update the management of many obstetric emergencies.

According to the results of other studies [14], student understanding of this topic did not significantly improve after clinical simulation. However, in a study with participants who were midwives, an improvement in understanding along with improved efficiency was observed compared with a group of midwives not completing the clinical simulation course [12].

For most of the questionnaire items related to the self-assurance and confidence of our participants, scores increased by a mean of more than 2.5 points. This has also been observed by others who observed the improved confidence of midwives assisting a simulation-based course on postpartum blood loss management, although the method used to assess confidence was not specified [15]. In our study, not only were participant replies collected adequately in terms of time and manner, but we also performed a comparative analysis and obtained statistical significance.

Among the limitations of this study is the drop out of some participants at 6 months post-workshop. This loss of students to follow up was likely due to a loss of motivation over time, or the lack of interest of a minority of students whose performance was below the average. In an analysis of the 25 students lost to follow up, it was observed that their mean MCT score at 2 weeks post-workshop was 7.8, while the mean for the whole group was 8.5.

Another limitation was the lack of a control group of students who did not complete the workshop. As the workshop was offered to all students in their fourth year of Medicine, a participation bias of the most applied students was assumed. We, nevertheless, consider the excellent participation rate of 97.2% a main strength of this study.

As only medical students were enrolled, we could not assess the role of this simulation-based workshop in improving the routine management of real patients with obstetric haemorrhage. This could be resolved if this workshop was offered to resident doctors, obstetricians or midwives. This issue was addressed in a rural hospital in Tanzania, and the authors concluded that clinical simulation can lead to a 38% reduction in the number of patients experiencing postpartum haemorrhage [16].

Among the study strengths, as far as we are aware, no published study has had the same objectives and methods as ours. We should also mention the larger sample size than described in most published reports [14,15], and highlight the excellent response shown by the students throughout the whole study period.

Finally, our students perceived the simulation-based workshop as useful and necessary. To our knowledge, these characteristics have not been assessed previously in similar studies in the field of obstetrics. We feel that exploring factors such as these is essential to determine the impact of this type of intervention on student behaviour and learning.

## 5. Conclusions

This simulation-based blood loss quantification workshop designed for medical students in their fourth year resulted in significant improvements in the theoretical–practical learning curve in the short term. Further improvements noted were the increased self-assurance and self-confidence of the students when facing this clinical situation. The workshop was also perceived as useful and necessary for their academic training. Based on these findings, we would recommend the use of more clinical reconstruction teaching interventions in faculties of medicine, as they seem much appreciated by students.

## Figures and Tables

**Table 1:** List of study variables along with their assessment method and qualifying measures.

Variable	Assessment Method	Qualifier
Sex	Student characteristic	Qualitative nominal
Age	Student characteristic	Quantitative discrete
Previous passive or active experience with clinical situations involving more than 1 L of blood loss	Student characteristic	Quantitative discrete 0 occasions; ≥1 occasion
Theoretical–practical knowledge	Multiple choice test	Quantitative discrete Score of 0–10 in 0.5-point steps
Self-assurance	Self-administered questionnaire	Quantitative discrete Likert scale (0–10) Poor (0–2), medium (3–4), good (5–6), very good (7–8) and excellent (9–10)
Self-confidence	Self-administered questionnaire	Quantitative discrete Likert scale (0–10) Not at all confident (0–2), scarcely confident (3–4), somewhat confident (5–6), confident (7–8) and very confident (9–10)
Perceived utility	Self-administered questionnaire	Quantitative discrete Likert scale (0–10) Not at all useful (0–2), not really useful (3–4), indifferent (5–6), useful (7–8) and definitely useful (9–10)
Feedback	Self-administered questionnaire	Three questions with different non-exclusive answers (students could mark as many options as they wished) Three questions with open answers

**Table 2:** MCT results and improvements during the study course.

Results of Multiple Choice Test							
	Pre-WS (N = 142)	Post-WS 2 Weeks (N = 137)	Short-Term Improvement (μ post—μ pre) (N = 137)	*p*	Post-WS 6 Months (N =111)	Long-Term Improvement (μ 6 mo—μ 2 wk) (N =111)	*p*
Score (/10)	7.47 ± 1.66	8.52 ± 1.06	1.01 ± 1.60 (8.52–7.47)	<0.05	7.47 ± 1.51	−1.15 ±1.24 (7.47–8.52)	<0.05

**Table 3:** Results of the self-assurance questionnaire and improvements during the study course. WS = workshop; wk = weeks; mo = months; BLQ = blood loss quantification.

Self-Assurance Questionnaire							
	Pre-WS (N = 141)	Post-WS 2 Weeks (N = 138)	Short-Term Improvement (μ post—μ pre) (N = 138)	*p*	Post-WS 6 Months (N = 123)	Long-Term Improvement (μ 6 mo—μ 2 wk) (N = 121)	*p*
1. Theoretical BLQ knowledge	4.69 ± 2.21	7.37 ± 1.34	2.68 (7.37–4.69)	<0.05	7.47 ± 1.38	0.02 ± 1.36 (7.47–7.37)	0.88
2. Practical BLQ knowledge	4.71 ± 2.27	7.72 ± 1.13	2.98 (7.72–4.71)	<0.05	7.58 ± 1.31	−0.18 ± 1.16 (7.58–7.72)	0.09
3. Practical management skills	2.79 ± 2.26	6.87 ± 1.44	4.10 (6.87–2.79)	<0.05	6.68 ± 1.53	−0.27 (6.68–6.87)	<0.05

**Table 4:** Results of the self-confidence questionnaire and improvements during the study course. WS = workshop; wk = weeks; mo = months; BLQ = blood loss quantification; PP = postpartum.

Self-Confidence Questionnaire							
	Pre-WS (N = 141)	Post-WS 2 Weeks (N = 138)	Short-Term Improvement (μ post—μ pre) (N = 138)	*p*	Post-WS 6 Months (N =123)	Long-Term Improvement (μ 6 mo—μ 2 wk) (N = 121)	*p*
4. Experience with BLQ	3.65 ± 2.3	6.58 ± 1.43	3.02 (6.59–3.57)	<0.05	6.45 ± 1.66	−0.29 (6.43–6.71)	<0.05
5. Controlling blood loss	2.82 ± 2.13	6.09 ± 1.58	3.29 (6.09–2.80)	<0.05	6.00 ± 1.79	−0.26 (5.98–6.24)	0.093
6. Controlling initial situation	2.94 ± 2.07	6.05 ± 1.68	3.18 (6.06–2.88)	<0.05	5.86 ± 1.84	−0.31 (5.84–6.15)	0.065
7. Visual BLQ	3.70 ± 2.20	6.40 ± 1.62	2.79 (6.39–3.60)	<0.05	6.46 ± 1.65	−0.11 (6.43–6.55)	0.457
8. Gravimetric BLQ	4.60 ± 2.51	7.91 ± 1.30	3.35 (7.92–4.57)	<0.05	7.24 ± 1.61	−0.76 (7.24–8.00)	<0.05
9. Differentiating between mild and severe blood loss	4.52 ± 2.22	7.30 ± 1.37	2.79 (6.39–3.60)	<0.05	7.11 ± 1.59	−0.23 (7.10–7.32)	0.10
10. Coordinating with other staff	4.84 ± 2.35	7.37 ± 1.43	2.58 (7.38–4.80)	<0.05	7.32 ± 1.57	−0.14 (7.30–7.44)	0.31
11. Preventing severe blood loss	3.28 ± 2.16	6.36 ± 1.77	3.11 (6.36–3.24)	<0.05	6.52 ± 1.60	0.11 (6.49–6.38)	0.52
12. Assisting a physician during blood loss	4.62 ± 2.44	7.20 ± 1.61	2.66 (7.22–4.56)	<0.05	6.99 ± 1.64	−0.36 (6.98–7.34)	<0.05
13. Managing blood loss under supervision of obstetrician	4.94 ± 2.46	7.25 ± 1.62	2.39 (7.26–4.88)	<0.05	7.10 ± 1.79	−0.28 (7.08–7.36)	0.10
14. Managing blood loss under supervision of a medical intern	4.55 ± 2.33	6.90 ± 1.66	2.42 (6.91–4.49)	<0.05	6.92 ± 1.80	−0.10 (6.89–6.99)	0.55
15. Managing blood loss without supervision	1.65 ± 1.90	4.93 ± 1.96	3.38 (4.94–1.57)	<0.05	4.59 ± 2.20	−0.48 (4.56–5.04)	<0.05

**Table 5:** Results of the perceived usefulness questionnaire and improvements during the study course. WS = workshop; wk= week; mo = month; BLQ = blood loss quantification.

Perceived Usefulness Questionnaire				
	Post-WS 2 Weeks (N = 138)	Post-WS 6 Months (N = 123)	Long-Term Improvement (μ 6 mo—μ 2 wk) (N = 121)	*p*
16. WS usefulness	9.14 ± 1.02	8.98 ± 1.19	−0.16 ± 1.19 (8.98–9.14)	0.13
17. Improved BLQ theoretical knowledge	8.97 ± 1.20	8.99 ± 1.11	0.06 ± 1.31 (8.98–8.93)	0.63
18. Improved BLQ practical knowledge	9.04 ± 1.06	8.68 ± 1.39	−0.34 ± 1.41 (8.67–9.02)	<0.05
19. Reduced stress when faced with blood loss	8.38 ± 1.30	8.07 ± 1.56	−0.34 ± 1.45 (8.06–8.39)	<0.05
20. WS needed in theoretical terms	8.96 ± 1.38	8.81 ± 1.57	−0.14 ± 1.82 (8.80–8.94)	0.40
21. WS needed in practical terms	9.31 ± 1.04	9.23 ± 1.11	−0.12 ± 1.08 (9.22–9.34)	0.24
22. WS should be obligatory	9.02 ± 1.39	8.85 ± 1.41	−0.16 ± 1.36 (8.84–8.99)	0.21

## Data Availability

The data used to support the findings of the present study are available from the corresponding author upon request.

## Appendix A. Self-Confidence, Self-Assurance, Usefulness and Feedback Questionnaires

*Self-assurance*										
	Question		Reply							
1.	**How would you describe your theoretical knowledge** **of blood loss quantification?**		Poor (0–2)		Medium (3–4)		Good (5–6)	Very good (7–8)		Excellent (9–10)
2.	**How would you describe your practical knowledge** **of blood loss quantification?**		Poor (0–2)		Medium (3–4)		Good (5–6)	Very good (7–8)		Excellent (9–10)
3.	**How would you describe your practical skills in managing obstetric blood loss?**		Poor (0–2)		Medium (3–4)		Good (5–6)	Very good (7–8)		Excellent (9–10)
*Self-confidence*										
	Question		Reply							
1.	**How confident are you at dealing with obstetric blood loss?**		Not at all (0–2)		Scarcely (3–4)		Somewhat (5–6)	Confident (7–8)		Very (9–10)
2.	**How confident are you at controlling blood loss?**		Not at all (0–2)		Scarcely (3–4)		Somewhat (5–6)	Confident (7–8)		Very (9–10)
3.	**How confident are you at controlling the initial situation?**		Not at all (0–2)		Scarcely (3–4)		Somewhat (5–6)	Confident (7–8)		Very (9–10)
4.	**How confident are you at quantifying blood loss visually (subjective)?**		Not at all (0–2)		Scarcely (3–4)		Somewhat (5–6)	Confident (7–8)		Very (9–10)
5.	**How confident are you at quantifying blood loss gravimetrically (objective)?**		Not at all (0–2)		Scarcely (3–4)		Somewhat (5–6)	Confident (7–8)		Very (9–10)
6.	**How confident are you at differentiating between mild and severe blood loss?**		Not at all (0–2)		Scarcely (3–4)		Somewhat (5–6)	Confident (7–8)		Very (9–10)
7.	**How confident are you at coordinating with the medical staff present?**		Not at all (0–2)		Scarcely (3–4)		Somewhat (5–6)	Confident (7–8)		Very (9–10)
8.	**How confident are you at preventing severe blood loss?**		Not at all (0–2)		Scarcely (3–4)		Somewhat (5–6)	Confident (7–8)		Very (9–10)
9.	**How confident would you feel assisting a clinician during a blood loss episode?**		Not at all (0–2)		Scarcely (3–4)		Somewhat (5–6)	Confident (7–8)		Very (9–10)
10.	**How confident would you feel assisting an obstetrician during a blood loss episode?**		Not at all (0–2)		Scarcely (3–4)		Somewhat (5–6)	Confident (7–8)		Very (9–10)
11.	**How confident would you feel under the supervision of a resident doctor?**		Not at all (0–2)		Scarcely (3–4)		Somewhat (5–6)	Confident (7–8)		Very (9–10)
12.	**How confident would you feel assisting a blood loss episode without supervision?**		Not at all (0–2)		Scarcely (3–4)		Somewhat (5–6)	Confident (7–8)		Very (9–10)
*Perceived usefulness*										
	Statement		Agreement level							
1.	**I found the workshop useful**		No, not at all (0–2)		No, not really (3–4)		Indifferent (5–6)	Yes (7–8)		Yes, definitely (9–10)
2.	**My theoretical knowledge of obstetric blood loss has improved**		No, not at all (0–2)		No, not really (3–4)		Indifferent (5–6)	Yes (7–8)		Yes, definitely (9–10)
3.	**My practical knowledge of obstetric blood loss has improved**		No, not at all (0–2)		No, not really (3–4)		Indifferent (5–6)	Yes (7–8)		Yes, definitely (9–10)
4.	**This workshop will reduce my stress levels when dealing with a blood loss episode in the future.**		No, not at all (0–2)		No, not really (3–4)		Indifferent (5–6)	Yes (7–8)		Yes, definitely (9–10)
5.	**This workshop is necessary to gain theoretical knowledge**		No, not at all (0–2)		No, not really (3–4)		Indifferent (5–6)	Yes (7–8)		Yes, definitely (9–10)
6.	**This workshop is necessary to gain practical knowledge**		No, not at all (0–2)		No, not really (3–4)		Indifferent (5–6)	Yes (7–8)		Yes, definitely (9–10)
7.	**This workshop should be obligatory for all medical undergraduates**		No, not at all (0–2)		No, not really (3–4)		Indifferent (5–6)	Yes (7–8)		Yes, definitely (9–10)
*Feedback*										
	Question	Reply (mark one or several)								
1.	**How did you feel about assisting the workshop?**	Curious		Unsure		Anxious			None of these	
2.	**How did you feel when conducting the simulation?**	Sure of yourself		Confused		Stressed/tense			None of these	
3.	**How do you feel about the feedback session?**	It was useful		It helped me connect with my peers and share our ideas		It was an opportunity to confront each other			None of these	
										
